# SNP rs322931 (C>T) in miR-181b and rs7158663 (G>A) in MEG3 aggravate the inflammatory response of anal abscess in patients with Crohn’s disease

**DOI:** 10.18632/aging.204014

**Published:** 2022-04-14

**Authors:** Chaoxiang Zhong, Qiuju Yao, Jing Han, Jie Yang, Fei Jiang, Qiong Zhang, Haiyi Zhou, Yuchao Hu, Wei Wang, Yan Zhang, Ye Sun

**Affiliations:** 1 Anorectal, Shuyang County’s Hospital of TCM, Shuyang Affiliated Hospital of Nanjing University of Traditional Chinese Medicine, Shuyang 223600, Jiangsu, China

**Keywords:** anal abscess, inflammation, MEG3, miR-181b, TNF

## Abstract

Background: The MEG3/miR-181b signaling has been implicated in the pathogenesis of several diseases including Crohn’s disease. This work aimed to study the correlation between SNPs in MEG3/miR-181b and the severity of anal abscess in patients with Crohn’s disease.

Methods: Quantitative real-time PCR was performed to analyze the expression of MEG3 and miR-181b. ELISA was carried out to examine the expression of TNF-α, IL-1β, IL-6, CRP, SSA, AAT, AAG and HPT in the peripheral blood of patients with Crohn’s disease. Luciferase assay was performed to explore the role of miR-181b in the expression of MEG3 and TNF-α.

Results: The expression of MEG3 and miR-181b in the peripheral blood of patients with Crohn’s disease was remarkably associated with the rs322931 and rs7158663 polymorphisms. rs322931 (C>T) in miR-181b and rs7158663 (G>A) in MEG3 significantly promoted the expression of TNF-α, IL-1β, IL-6, CRP, SSA, AAT, AAG and HPT. Luciferase assay demonstrated that miR-181b was capable of repressing the expression of MEG3 and TNF-α through binding to their specific binding sites. Moreover, alteration of MEG3 and miR-181b expression also showed a remarkable impact on the MEG3/miR-181b/TNF-α signaling pathway in THP-1 cells.

Conclusions: In conclusion, our study demonstrated that two SNPs, rs322931 (C>T) in miR-181b and rs7158663 (G>A) in MEG3, could aggravate the inflammatory response of anal abscess in patients with Crohn’s disease via modulating the MEG3/miR-181b/TNF-α signaling pathway.

## INTRODUCTION

Anal abscess (AA) is among one of most commonly diagnosed anorectal conditions, and AA commonly develops in young males with an age of between 30 and 50 years old. The abscess is often derived from cryptoglandular infections of the proctodeal gland located in the inter-sphincteric area. AA cases are actually divided into several categories including perianal, inter-sphincteric, ischiorectal, as well as supralevator AAs based upon their location [1]. The annual incidence of AA can be up to 20 per 100000 people, while the chance of succeeding fistula can be up to 15% [2, 3]. So far, multiple factors have been reported to increase the risk of AA, such as obesity, diabetes, alcohol abuse, smoking, high salt consumption, as well as psychosocial stress [3–5].

Long noncoding RNA (lncRNA) belongs to a family of RNA molecules with no protein coding ability that contain at least 200 nucleotides [6]. An lncRNA named maternally conveyed gene 3 (MEG3) is an lncRNA that functions as a tumor suppressor in several types of cancers. For instance, MEG3 can inhibit the invasion as well as proliferation of cancer cells through enhancing EZH2 ubiquitination in gallbladder cancer cells [7]. MEG3 additionally inhibits the growth of liver cancer through decreasing the level of β-catenin via PKM2/PTEN signaling [8]. MEG3 also enhances the fibrosis as well as inflammatory responses of MCs, and the overexpression of MEG3 enhances the level of expression of proteins related to fibrosis as well as cytokines related to inflammation. On the other hand, the silencing of MEG3 inhibited fibrosis as well as the level of inflammation. The family of miR-181 miRNAs was found to participate in many vital biological functions in the onset of glioma. In particular, as a tumor suppressor, miRNA miR-181b is lowly expressed in glioma cells [9]. In addition, miR-181b was shown to influence the sensitivity of glioma cells to chemotherapy [10]. As a result, it was suggested that miR-181 expression can be used as a biomarker to predict chemotherapy response of glioblastoma patients [11]. The family of miR-181 miRNAs plays an essential role in the inflammation in the cardiovascular system, as well as in the formation of atherosclerotic plaques and acute strokes [12–14]. Simultaneously, the inflammatory factor levels in the serum of patients in the AS group were dramatically increased, indicating that miR-181b can play a pro-inflammatory role in endothelial cells to induce AS plaque formation by promoting the adhesion of monocytes while activating the synthesis of inflammatory chemokines as well as cytokines.

A recent study on the distinctions of gene expression in subjects with favorable emotional experiences (PE) identified a single nucleotide polymorphism (SNP) of rs322931 related to PE, and the SNP was also related to the reduced negative emotion as well as improved resilience, indicating a potential role of this SNP conferring psychopathological risks [15]. In another research, it was hypothesized that the rs322931 SNP may increase IS risk by reducing miR-181b expression, and the results confirmed for the first time that the CT/TT genotype of the rs322931 SNP in miR-181b acted as an independent factor for the risk of IS in both the analysis on single site association as well as the analysis of logistic regression [15]. Past predictions on SNP functions showed that the rs7158663 as well as rs4081134 SNP in MEG3 is positioned in the binding site of transcription factors [16]. For that reason, it was speculated that the rs4081134 as well as rs7158663 SNPs can change lncRNAMEG3 expression to reduce lung cancer risks. Cao et al. genotyped 5 SNPs, i.e., rs4081134, rs11160608, rs3087918, rs10144253, as well as rs7158663, located in MEG3 to study their roles in the onset of colorectal cancer, and found that the rs7158663 SNP of MEG3 was linked to the risk of colorectal cancer in Chinese [17].

In previous researches, MEG3 has been implicated as a key regulator in the pathogenesis of human gastric carcinogenesis by modulating the signaling between miR-181 and Bcl2 [18]. And in other diseases such as IS (ischemic stroke), the signaling of MEG3/miR-181b, as well as the association between the polymorphisms located in MEG3/miR-181 and the risk of IS, was also investigated. Accordingly, it was found that the G allele of rs7158663 in MEG3 and the T allele of rs322931 in miR-181b was associated with higher risk of IS [19]. Moreover, the production of tumor necrosis factor-α (TNF-α) was also reported to be associated with more intense inflammatory activity in patients with Crohn’s disease [20]. Therefore, we hypothesized that the combination of SNP rs322931 (C>T) in miR-181b and rs7158663 (G>A) in MEG3 would also regulate the inflammatory response of anal abscess in patients with Crohn’s disease. In this study, by recruiting patients with Crohn’s disease, we compared the parameters including MEG3, miR-181b, and TNF expression as well as the levels of inflammatory factors in patients carrying different genotype of MEG3 and miR-181b, so as to study the correlation between the SNPs and MEG3/miR-181b expression as well as the severity of anal abscess in patients with Crohn’s disease.

## RESULTS

### Patient characterization

In this study, we recruited 206 patients with Crohn’s disease and collected their peripheral blood samples. Genotyping was performed to decide the genotypes of rs322931 and rs7158663 in all of the patients. These patients were divided into four groups according to their genotypes at rs322931 and rs7158663: 1, rs7158663 GG/rs322931 CC (N=76); 2, rs7158663 AG+AA/rs322931 CC (N=62); 3. rs7158663 GG/rs322931 CT+TT (N=36); 4. rs7158663 AG+AA/rs322931 CT+TT (N=32). The information of the patients including their age, sex, disease localization, disease duration and CDAI was summarized in Table 1. Student’s t test indicated that no obvious difference was observed for all above characters in the three groups.

**Table 1:** Patient information of recruited patients with Crohn’s disease.

**Characteristics**	**Rs7158663 GG/Rrs322931 CC (N=76)**	**Rs7158663 AG+AA / Rrs322931 CC (N=62)**	**Rs7158663 GG / Rrs322931 CT+TT** **(N=36)**	**Rs7158663 AG+AA / Rrs322931 CT+TT** **(N=32)**	***P* value**
Sex, female/male	42/34	38/24	20/16	20/12	0.522
Age, years	35.6 ± 5.4	36.6 ± 5.3	35.1 ± 7.1	33.1 ± 6.0	0.312
Disease localization					
Ileocecal, n (%)	25 (32.9)	23 (37.1)	15 (41.7)	15 (46.9)	0.336
Colonic, n (%)	23 (37.1)	23 (37.1)	12 (33.3)	11 (34.4)	0.822
Ileocolic, n (%)	28 (30.0)	16 (25.8)	9 (25.0)	6 (18.7)	0.260
Disease duration, years	5.8 ± 2.3	6.2 ± 2.8	5.9 ± 3.1	4.0 ± 0.4	0.626
CDAI	223.5 ± 35.6	216.0 ± 66.5	235.8 ± 23.9	225.7 ± 22.9	0.481

### Different expression of MEG3 and miR-181b in the peripheral blood of Crohn’s disease patients carrying different genotypes of rs322931 and rs7158663

**Figure 1:**
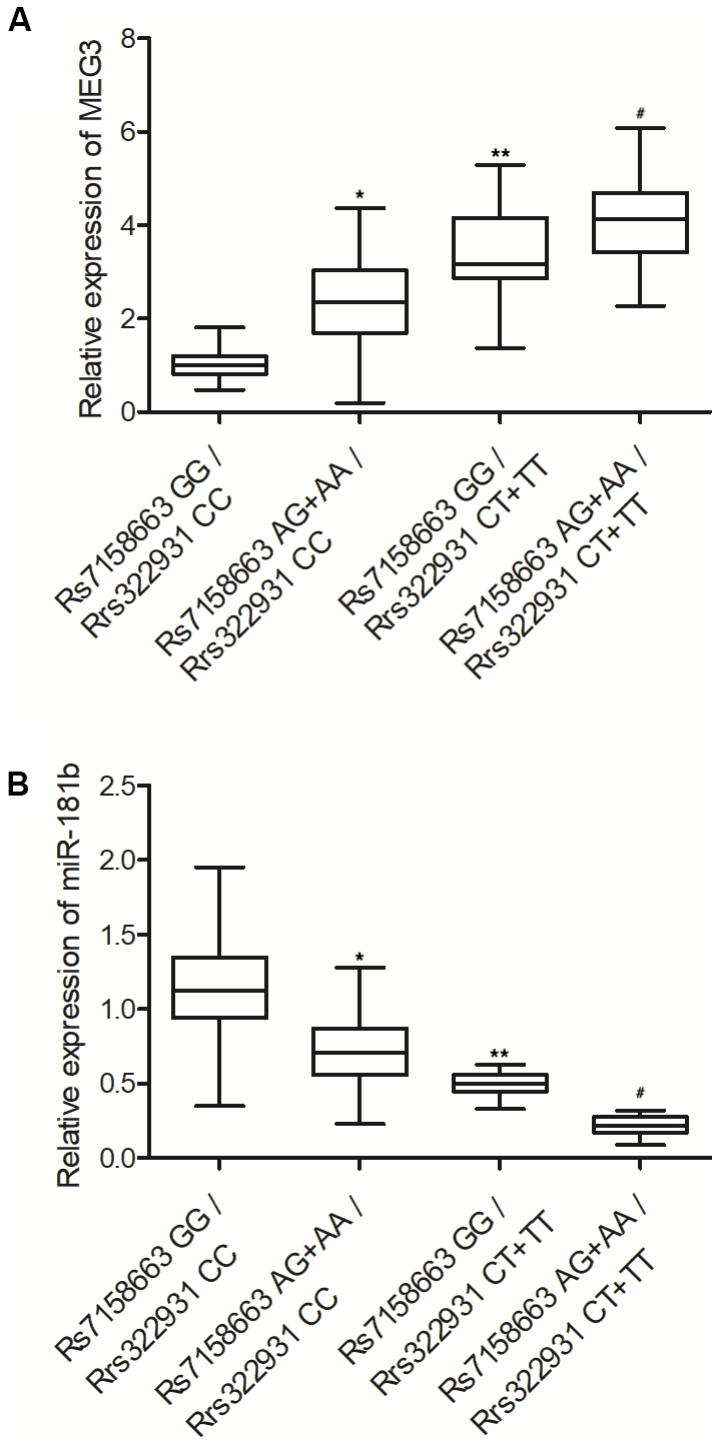
The expression of MEG3 (**A**) was progressively elevated while the expression of miR-181b was progressively suppressed (**B**) in the peripheral blood of Crohn’s disease patients carrying rs7158663 GG/rs322931 CC (N=76), rs7158663 AG+AA/rs322931 CC (N=62), rs7158663 GG/rs322931 CT+TT (N=36), and rs7158663 AG+AA/rs322931 CT+TT (N=32) genotypes (* P value < 0.05 vs. rs7158663 GG/rs322931 CC group; ** P value < 0.05 vs. rs7158663 AG+AA/rs322931 CC group; # P value < 0.05 vs. rs7158663 GG/rs322931 CT+TT group; 3 biological repeats).

In order to compare the different expression of MEG3 and miR-181b in the peripheral blood collected from patients carrying different genotypes, qPCR was performed to analyze the gene expression of MEG3 and miR-181b in the peripheral blood samples. Obviously, the expression of MEG3 was progressively elevated in the peripheral blood from patients with rs7158663 GG/rs322931 CC, rs7158663 AG+AA/rs322931 CC, rs7158663 GG/rs322931 CT+TT, and rs7158663 AG+AA/rs322931 CT+TT (Figure 1A), whereas the expression of miR-181b showed the opposite trend in the four groups (Figure 1B). These results indicated that rs7158663 GG and rs322931 CC were negatively correlated with the expression of MEG3 but positively correlated with the expression of miR-181b in the peripheral blood of patients with Crohn’s disease.

### Rs7158663 AG+AA/rs322931 CT+TT were positively correlated with the expression of TNF-α, IL-1β and IL-6

**Figure 2:**
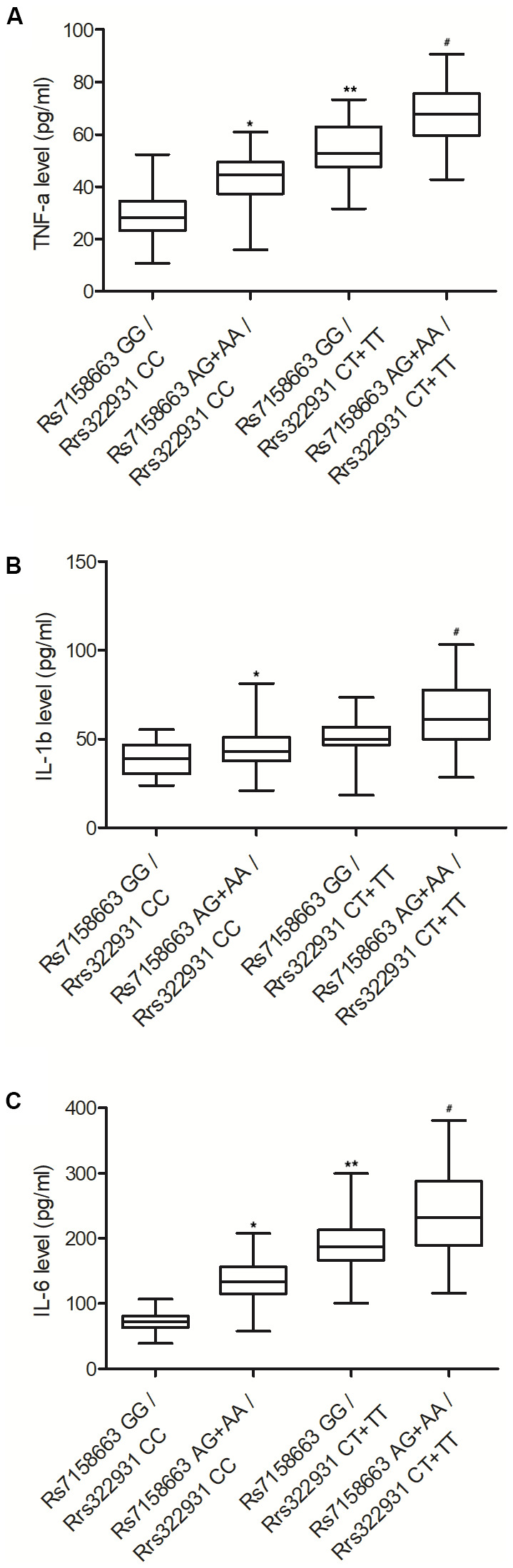
The expressions of TNF-α (**A**), IL-1β (**B**) and IL-6 (**C**) were all progressively elevated in the peripheral blood of Crohn’s disease patients carrying rs7158663 GG/rs322931 CC (N=76), rs7158663 AG+AA/rs322931 CC (N=62), rs7158663 GG/rs322931 CT+TT (N=36), and rs7158663 AG+AA/rs322931 CT+TT (N=32) genotypes (* P value < 0.05 vs. rs7158663 GG/rs322931 CC group; ** P value < 0.05 vs. rs7158663 AG+AA/rs322931 CC group; # P value < 0.05 vs. rs7158663 GG/rs322931 CT+TT group; 3 biological repeats).

To examine whether rs7158663 and rs322931 polymorphisms correlated with the expression of TNF-α, IL-1β and IL-6, ELISA was carried out to compare the abundance of TNF-α, IL-1β and IL-6 protein in the peripheral blood of Crohn’s disease patients carrying different genotypes at rs7158663 and rs322931. The protein expression of TNF-α (Figure 2A), IL-1β (Figure 2B) and IL-6 (Figure 2C) showed a progressively increasing trend in the peripheral blood collected from patients with rs7158663 GG/rs322931 CC, rs7158663 AG+AA/rs322931 CC, rs7158663 GG/rs322931 CT+TT, and rs7158663 AG+AA/rs322931 CT+TT genotypes.

### Rs7158663 AG+AA/rs322931 CT+TT were positively correlated with the expression of CRP and SSA

**Figure 3:**
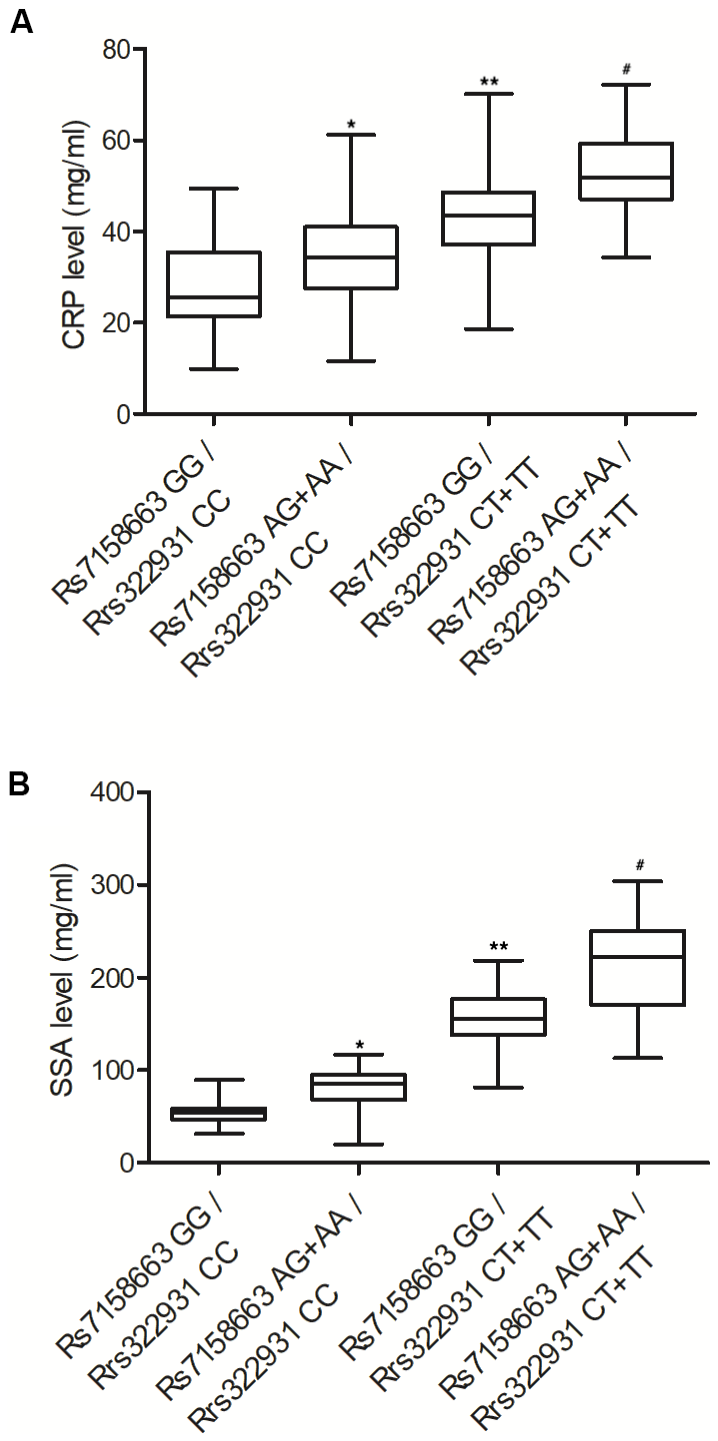
The expressions of CRP (**A**) and SSA (**B**) were both progressively elevated in the peripheral blood of Crohn’s disease patients carrying rs7158663 GG/rs322931 CC (N=76), rs7158663 AG+AA/rs322931 CC (N=62), rs7158663 GG/rs322931 CT+TT (N=36), and rs7158663 AG+AA/rs322931 CT+TT (N=32) genotypes (* P value < 0.05 vs. rs7158663 GG/rs322931 CC group; ** P value < 0.05 vs. rs7158663 AG+AA/rs322931 CC group; # P value < 0.05 vs. rs7158663 GG/rs322931 CT+TT group; 3 biological repeats).

In order to check whether the expression of CRP and SSA was affected by rs7158663 and rs322931 polymorphisms, the abundance of CRP and SSA protein in the peripheral blood of patients of Crohn’s disease was measured using ELISA. An apparent elevation was observed in the protein expression of CRP (Figure 3A) and SSA (Figure 3B) in the peripheral blood of patients carrying rs7158663 GG/rs322931 CC, rs7158663 AG+AA/rs322931 CC, rs7158663 GG/rs322931 CT+TT, and rs7158663 AG+AA/rs322931 CT+TT genotypes.

### Rs7158663 AG+AA/rs322931 CT+TT were positively correlated with the expression of AAT, AAG and HPT

**Figure 4:**
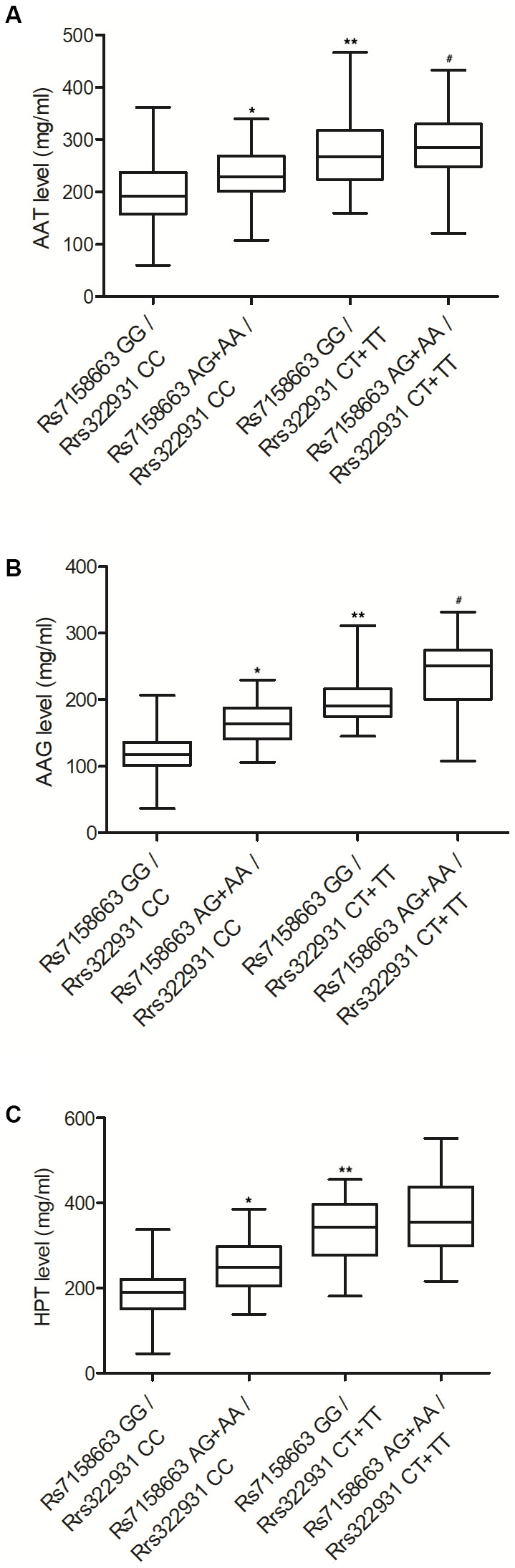
The expressions of AAT (**A**), AAG (**B**) and HPT (**C**) were all progressively elevated in the peripheral blood of Crohn’s disease patients carrying rs7158663 GG/rs322931 CC (N=76), rs7158663 AG+AA/rs322931 CC (N=62), rs7158663 GG/rs322931 CT+TT (N=36), and rs7158663 AG+AA/rs322931 CT+TT (N=32) genotypes (* P value < 0.05 vs. rs7158663 GG/rs322931 CC group; ** P value < 0.05 vs. rs7158663 AG+AA/rs322931 CC group; # P value < 0.05 vs. rs7158663 GG/rs322931 CT+TT group; 3 biological repeats).

Moreover, the protein expression of AAT, AAG and HPT was also analyzed in the peripheral blood of patients carrying different genotypes at rs7158663 and rs322931 to verify their effects on the expression of AAT, AAG and HPT. Similar to the results of TNF-α, IL-1β, IL-6, CRP and SSA, the expression of AAT (Figure 4A), AAG (Figure 4B) and HPT (Figure 4C) also showed a progressively increasing trend in the peripheral blood of patients carrying rs7158663 GG/rs322931 CC, rs7158663 AG+AA/rs322931 CC, rs7158663 GG/rs322931 CT+TT, and rs7158663 AG+AA/rs322931 CT+TT alleles.

### MiR-181b suppressed the expression of MEG3 and TNF-α through binding to their 3’ UTR

**Figure 5:**
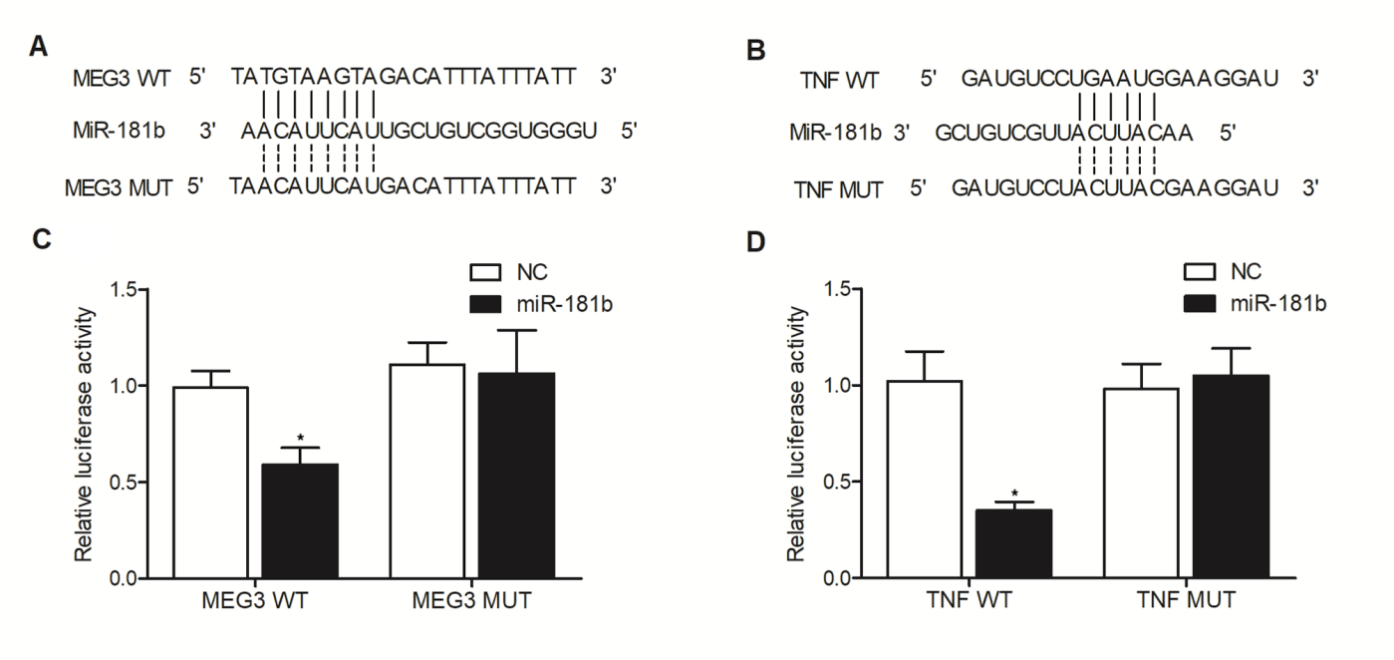
Sequence analysis indicated the binding of miR-181b to the 3’ UTR of MEG3 (**A**) and TNF-α (**B**), respectively. And the luciferase activity of wild-type MEG3 (**C**) as well as TNF-α (**D**) was suppressed by miR-181b in THP-1 cells, thus verifying that miR-181b could suppress the expression of MEG3 and TNF-α through binding to their 3’ UTR (* P value < 0.05 vs. NC group; 3 biological repeats).

Binding site screening showed that miR-181b could potentially target the 3’ UTRs of MEG3 (Figure 5A) and TNF-α (Figure 5B). In order to verify the inhibitory role of miR-181b in the expression of MEG3 and TNF-α mRNA, luciferase vectors containing wild type and mutant MEG3 and TNF-α were established and transfected into THP-1 cells along with miR-181b. The luciferase activities of wild-type MEG3 (Figure 5C) and TNF-α 3’UTR (Figure 5D) were significantly suppressed by miR-181b, while the luciferase activities of mutant MEG3 and TNF-α 3’UTR were not regulated. These results indicated that miR-181b was capable of repressing the expression of MEG3 and TNF-α mRNA through binding to their 3’ UTR.

### MEG3 siRNA and miR-181b precursors suppressed the expression of MEG3 and TNF-α but activated the expression of miR-181b

**Figure 6:**
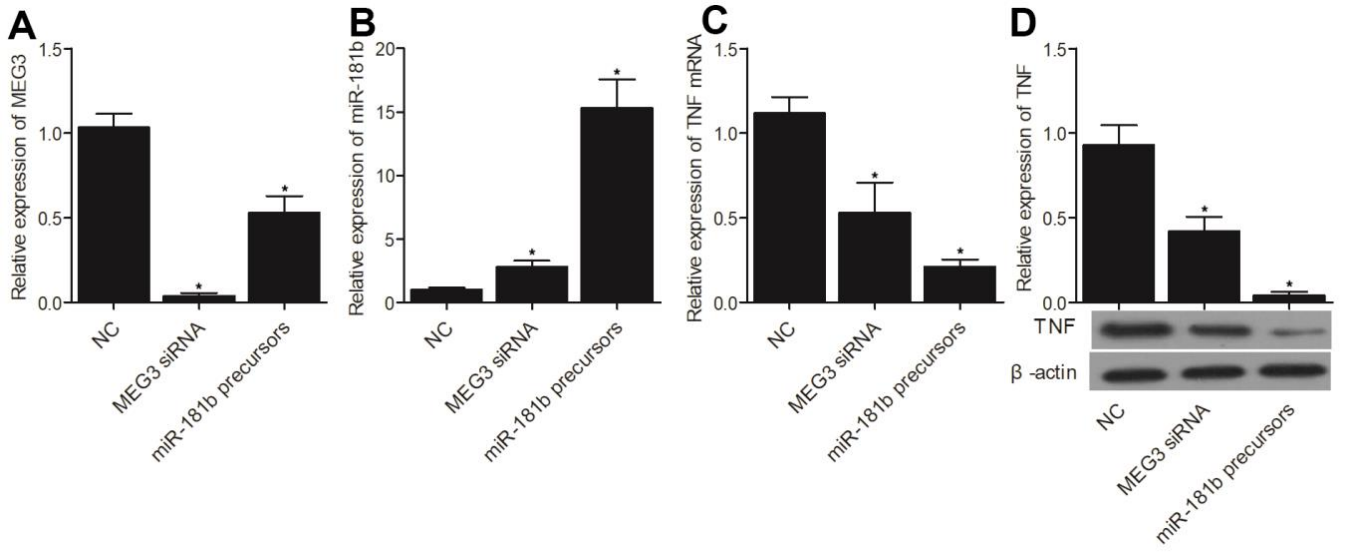
The successful transfection of MEG3 siRNA, which was validated by the evidently reduced expression of MEG3 (**A**), promoted the expression of miR-181b (**B**) and repressed the expression of TNF-α mRNA (**C**) and protein (**D**). Moreover, the successful transfection of miR-181b precursors, which was validated by the significantly promoted miR-181b expression (**B**), suppressed the expression of MEG3 (**A**), TNF-α mRNA (**C**) and protein (**D**) (* P value < 0.05 vs. NC group; 3 biological repeats).

To further explore the molecular mechanism underlying the regulatory relationship between MEG3 and miR-181b, MEG3 siRNA and miR-181b precursors were transfected into THP-1 cells, and qPCR was carried out to measure the expression of MEG3, miR-181b and TNF-α mRNA, while Western blot was used to analyze the expression of TNF-α protein. The expression of MEG3 (Figure 6A), as well as the expression of TNF-α mRNA (Figure 6C) and TNF-α protein (Figure 6D), was remarkably suppressed by MEG3 siRNA, whereas the expression of miR-181b (Figure 6B) was notably enhanced by MEG3 siRNA. It is worth noting that the efficiency of miR-181b precursors on inhibiting the expression of MEG3 and TNF-α mRNA/protein was significantly stronger than that of MEG3 siRNA.

### P-MEG3 and p-anti-miR-181b enhanced the expression of MEG3 and TNF-α but inhibited the expression of miR-181b

**Figure 7:**
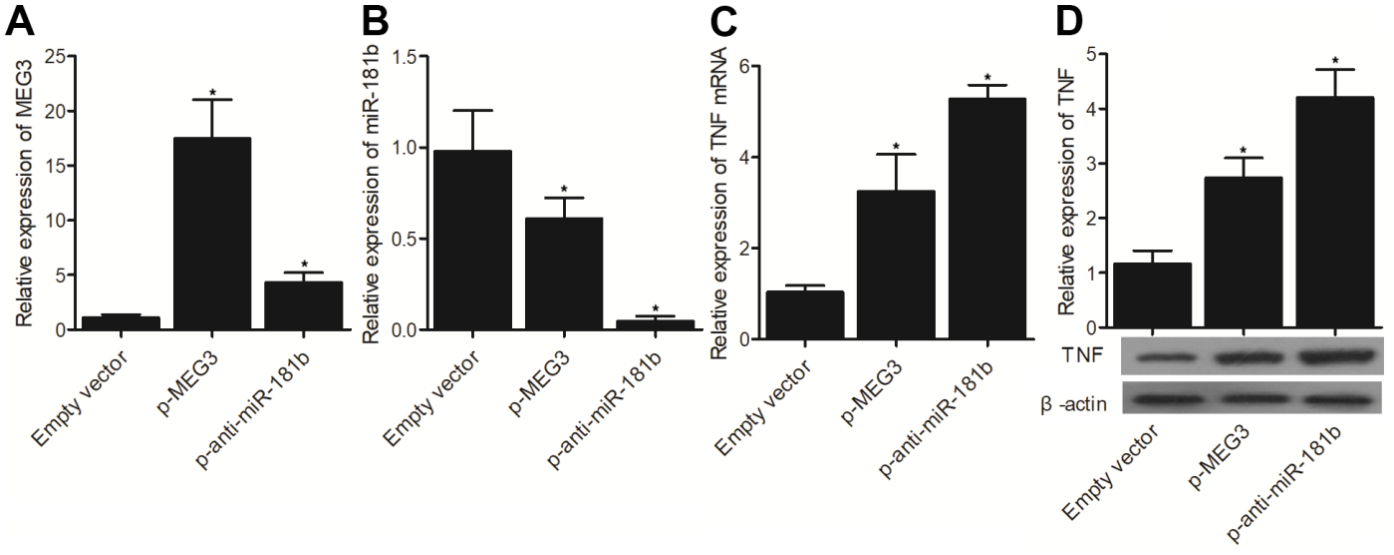
The successful transfection of p-MEG3, which was validated by the evidently enhanced expression of MEG3 (**A**), inhibited the expression of miR-181b (**B**) and increased the expression of TNF-α mRNA (**C**) and protein (**D**). Moreover, the successful transfection of p-anti-miR-181b, which was validated by the evident inhibition of miR-181b expression (**B**), promoted the expressions of MEG3 (**A**), TNF-α mRNA (**C**) and protein (**D**) (* P value < 0.05 vs. Empty vector group; 3 biological repeats).

Furthermore, we transfected p-MEG3 and p-anti-miR-181b into THP-1 cells to examine their effect on MEG3, miR-181b and TNF-α expression. P-MEG3 and p-anti-miR-181b apparently enhanced the expression of MEG3 (Figure 7A), as well as the expression of TNF-α mRNA (Figure 7C) and TNF-α protein (Figure 7D). The expression of miR-181b was evidently suppressed by p-MEG3 and p-anti-miR-181b (Figure 7B).

## DISCUSSION

In this study, we analyzed the expression of MEG3 and miR-181b in the peripheral blood of patients of Crohn’s disease to explore the effect of rs7158663 and rs322931. The expression of MEG3 was progressively elevated in the peripheral blood from patients carrying rs7158663 GG/rs322931 CC, rs7158663 AG+AA/rs322931 CC, rs7158663 GG/rs322931 CT+TT, and rs7158663 AG+AA/rs322931 CT+TT genotypes, whereas the expression of miR-181b showed the opposite trend in the four groups. The G > A transition in the rs7158663 SNP of MEG3 is related to elevated risks of colon cancer [17]. Likewise, Ghaedi et al. stated that the AA allele of rs7158663 in MEG3 was associated with a considerable rise in the susceptibility to diabetes [21]. Furthermore, this SNP might change the local folding structure of RNA to obstruct the interactions between miRNAs and lncRNAs [21]. Moreover, the over-expression of MEG3-4 can result in elevated levels of inflammatory responses, more significant lung injuries, as well as elevated mortality, suggesting an important role of this lncRNA in inflammation regulation [22]. A bioinformatic study also showed the potential binding between MEG3 and miR-181a, indicating that MEG3 can interact with miR-181a by direct targeting [23]. In this study, we carried out luciferase assay to explore the inhibitory role of miR-181b in MEG3 and TNF-α expression. The luciferase activities of wild type MEG3 and TNF-α were remarkably suppressed by miR-181b.

It was reported that miR-181b acts as a tumor suppressor to inhibit tumor cell proliferation while inhibiting its apoptosis in glioma [9, 11]. On top of that, miR-181b was shown to regulate the sensitivity of tumor cells to chemotherapy in many kinds of cancers, such as breast cancer, lung cancer, hepatocellular carcinoma, as well as colon cancer [24–26]. MiR-181b was also shown to act as a key member in the positive feedback loop linking cellular transformation to inflammation [27, 28]. It was revealed that the overexpression of miR181b reduced the apoptotic level of HK-2 cells induced by hypoxia, and suppressed the increased expression of TNF-α induced by hypoxia [29]. In this study, we transfected MEG3 siRNA/p-MEG3 and miR-181 precursors/anti-miR-181b into THP-1 cells to examine their effect on the expression of MEG3, miR-181b and TNF-α. MEG3 siRNA and miR-181b precursors significantly suppressed the expression of MEG3 and TNF-α, but enhanced the expression of miR-181b, whereas p-MEG3 and p-anti-miR-181b notably enhanced the expression of MEG3 and TNF-α but repressed the expression of miR-181b. On top of that, miR-181a was a key member in the neurocircuitry of reward because miR-181a expression in hippocampal nerve cells was actually induced by dopamine and amphetamine signaling [30, 31]. It was also postulated that the rs322931 SNP exerts a positive effect on the reward neurocircuitry by impacting the expression of miR-181a [32].

The TNF cytokine exerts a great impact on many biological processes. For example, low yet persistent TNF expression was revealed to induce rheumatoid arthritis as well as inflammatory bowel diseases (IBDs). Thus, TNF inhibitors may be a good candidate for patients who have failed first-line therapies [33]. A past study once showed that miR-124 expression was low in AA patients, along with substantially increased levels of IFN-γ, TNF-α, and IL-4. For that reason, the expression levels of miR-124 as well as related inflammatory factors may be used as biomarkers of AA. In this study, we performed ELISA to analyze the expression of TNF-α, IL-1β, IL-6, CRP, SSA, AAT, AAG and HPT in the peripheral blood of patients carrying different genotypes at rs7158663 and rs322931. The expression of TNF-α, IL-1β, IL-6, CRP, SSA, AAT, AAG and HPT was progressively elevated in the peripheral blood from patients carrying rs7158663 GG/rs322931 CC, rs7158663 AG+AA/rs322931 CC, rs7158663 GG/rs322931 CT+TT, and rs7158663 AG+AA/rs322931 CT+TT genotypes.

## CONCLUSIONS

In conclusion, our study demonstrated that the two SNPs, rs322931 (C>T) in miR-181b and rs7158663 (G>A) in MEG3, could aggravate the inflammatory response of anal abscess in patients with Crohn’s disease, via modulating the MEG3/miR-181b/TNF signaling pathway. The presence of minor allele rs322931 and rs7158663 could increase the MEG3 expression, thus resulting in the up-regulated expression of TNF via inhibiting the expression of miR-181b.

## MATERIALS AND METHODS

### Human subjects and sample collection

In this study, we recruited 206 patients with Crohn’s disease (CD) and collected their peripheral blood samples. Genotyping was performed to decide the genotypes of rs322931 and rs7158663 in all of the patients. These patients were then divided into four groups according to their genotypes at rs322931 and rs7158663, i.e., 1. rs7158663 GG/rs322931 CC group (N=76); 2. rs7158663 AG+AA/rs322931 CC group (N=62); 3. rs7158663 GG/rs322931 CT+TT group (N=36); and 4. rs7158663 AG+AA/rs322931 CT+TT group (N=32). The information of all patients including their age, sex, disease localization, disease duration and CDAI was collected, and the results of different groups were compared using Student’s t tests. More specifically, the medical diagnosis as well as the severity of CD was achieved based on the clinical, radiological, endoscopic, as well as histopathological results of the patients, and evaluated based on a standard method described elsewhere. The professional task of Compact Disc clients was assessed using the Crohn's Disease Activity Mark (CDAI). The Crohn’s Disease Activity Index was used in this study to assess the clinical activities of all CD patients. Institutional ethical committee has approved the protocol of this study.

### Genotyping by direct sequencing

In this study, genotyping was performed to decide the genotypes of rs322931 and rs7158663 in all of the patients enrolled in study. First, the genomic DNA was extracted from each collected peripheral blood sample by using a DNeasy extraction assay kit (Qiagen, Germantown, MD, USA) following the suggested standard assay protocol provided by the assay kit manufacturer on the instructions of the assay kit. The quality and concentration of extracted genomic DNA were measured by using a Nano Drop 2000 spectrometer (Thermo Fisher Scientific, Waltham, MA, USA) following the instrument manual. Then, 100 μl of each extracted genomic DNA sample at a concentration of 50 ng/μl were added into a 96-well plate and assayed using a direct-sequencing based Affymetrix Genome-wide HUMAN SNP Array 6.0 assay kit (Affymetrix, Thermo Fisher Scientific, San Jose, CA, USA) following the suggested standard assay protocol provided by the assay kit manufacturer on the instructions of the assay kit.

### RNA isolation and real-time PCR

In this study, qPCR was performed to analyze the expression of MEG3 and miR-181b in the peripheral blood samples. In brief, total RNA content was first isolated from the peripheral blood samples by making use of a miRNeasy miRNA isolation Mini assay kit (Qiagen, Valencia, CA, USA) following the suggested standard assay protocol provided by the assay kit manufacturer on the instructions of the assay kit. Then, the isolated RNA was reverse transcribed into cDNA by using a miRCURY universal RT assay kit (Exiqon, Woburn, MA, USA) following the suggested standard assay protocol provided by the assay kit manufacturer on the instructions of the assay kit. In the next step, the cDNA was used as the template to carry out real time PCR on a Prism 7900 HT real time PCR machine (ABI, Foster City, CA, USA) using a SYBR Green real time PCR Supermix (Thermo Fisher Scientific, Waltham, MA, USA) following the suggested standard assay protocol provided by the assay kit manufacturer on the instructions of the assay kit to determine the relative expression of MEG3, miR-181b, and TNF-α in each sample. The calculation was performed by utilizing the 2^−ΔΔCT^ approach with the U6 housekeeping gene as the internal standard.

### Cell culture and transfection

In this study, THP-1 cells were cultivated in a DMEM medium (Gibco, Thermo Fisher Scientific, Waltham, MA, USA) supplemented along with 10% of FBS (Gibco, Thermo Fisher Scientific, Waltham, MA, USA) and appropriate concentrations of penstrep double antibiotics (Sigma Aldrich, St. Louis, MO, USA). The culture was carried out under standard mammalian cell culture conditions of 37° C, 5% CO2 and saturated humidity. When the THP-1 cells reached 70% confluence, they were divided into 2 cell models. In cell model I, the THP-1 cells were divided into 3 groups, i.e., 1. NC group (THP-1 cells transfected with a negative control siRNA); 2. MEG3 siRNA group (THP-1 cells transfected with MEG3 siRNA); and 3. miR-181b precursor group (THP-1 cells transfected with miR-181b precursors). In cell model II, the THP-1 cells were also divided into 3 groups, i.e., 1. Empty vector group (THP-1 cells transfected with an empty vector); 2. p-MEG3 group (THP-1 cells transfected with a vector carrying MEG3); and 3. p-anti-miR-181b group (THP-1 cells transfected with a vector carrying anti-miR-181b). The transfection of both cell models was done using Lipofectamine 2000 (Invitrogen, Carlsbad, CA, USA) following the suggested transfection assay protocol provided by the transfection reagent manufacturer on the instructions. The cells were harvested after 48 h of transfection to determine the expression of target genes.

### Vector construction, mutagenesis and luciferase assay

The results of our binding site screening showed that miR-181b could potentially target the 3’ UTRs of MEG3 and TNF-α. In order to verify the inhibitory role of miR-181b in the expression of MEG3 and TNF-α, luciferase vectors containing wild type and mutant MEG3 and TNF-α were established. In brief, the wild type sequences of 3’ UTRs of MEG3 and TNF-α containing the miR-181b binding sites were first cloned into pGL luciferase vectors (Promega, Madison, WI, USA) to generate wild type MEG3 and TNF-α plasmids. In addition, site directed mutagenesis was carried out by using a Quick Change mutagenesis kit (Stratagene, San Diego, CA, USA) following the suggested standard assay protocol provided by the assay kit manufacturer to generate site-directed mutations in the miR-181b binding sites located in the 3’ UTRs of MEG3 and TNF-α, and the mutant type sequences of 3’ UTRs of MEG3 and TNF-α were also cloned into pGL luciferase vectors to generate mutant type MEG3 and TNF-α plasmids. Then, both wild type and mutant MEG3 and TNF-α vectors were co-transfected into THP-1 cells along with miR-181b using Lipofectamine 2000, and the luciferase activities of transfected cells were analyzed 48 h later by using Bright-Glo luciferase assay kit (Promega, Madison, WI, USA) following the suggested standard assay protocol provided by the assay kit manufacturer on the instructions of the assay kit.

### Western blot analysis

To determine the protein expression of TNF-α in each cell and tissue sample, the total protein in each cell and tissue sample was extracted by using a RIPA lysis buffer (Sigma Aldrich, St. Louis, MO, USA) following the suggested standard assay protocol provided by the reagent manufacturer, and the extracted protein was quantified by utilizing a BCA assay kit (Pierce, Rockford, IL) following the suggested standard assay protocol provided by the assay kit manufacturer on the instructions of the assay kit. Then, an appropriate amount of protein from each sample was resolved by using a 10% SDS gel under denaturing conditions, transferred onto an Immobilon polyvinylidene difluoride membrane (Millipore, Bedford, MA, USA), and probed with primary anti-TNF-α antibodies and corresponding secondary HRP-labeled anti-TNF-α antibodies following the suggested antibody incubation protocol provided by the antibody manufacturer (Abcam, Cambridge, MA, USA). In the next step, the blotted membrane was developed by using an ECL assay kit (Pierce, Rockford, IL, USA) following the suggested standard assay protocol provided by the assay kit manufacturer on the instructions of the assay kit and then visualized by using an Odyssey image system (Li-Cor Biosciences, Lincoln, NE, USA) following the operating manual of the system to determine the relative protein expression of TNF-α in each cell and tissue sample.

### ELISA

In this study, the levels of TNF-α, IL-1β, IL-6, CRP, SSA, AAT, AAG as well as HPT in each collected peripheral blood sample were assayed using ELISA assay kits (Diaclone Analysis, Besancon, France) following the suggested standard assay protocol provided by the assay kit manufacturer on the instructions of the assay kits.

### Statistical analysis

All data in this study was shown using mean ± SD. The comparison of two different groups was carried out by using Student’s ***t*** tests. And the comparison between multiple groups was done by one-way ANOVA with Tukey’s test being used as the post hoc test. All observations were repeated biologically for 3 times. The level of significance for statistical differences was set to P value < 0.05. All statistical analysis were done using GraphPad Prism (GraphPad, San Diego, CA, USA).

### Availability of data and material

The data that support the findings of this study are available from the corresponding author upon reasonable request.
